# Antimycobacterial Activity of *Hedeoma drummondii* against *Mycobacterium tuberculosis* and Non-Tuberculous Mycobacteria

**DOI:** 10.3390/antibiotics12050833

**Published:** 2023-04-29

**Authors:** Carmen Molina-Torres, Carlos Pedraza-Rodríguez, Lucio Vera-Cabrera, Jorge Ocampo-Candiani, Catalina Rivas-Morales, Ezequiel Viveros-Valdez

**Affiliations:** 1 Servicios de Dermatología, Hospital Universitario “José E. González”, Universidad Autónoma de Nuevo León (UANL), Madero y Gonzalitos, Col. Mitras Centro, Monterrey 66640, NL, Mexico; carmen.molinatr@uanl.edu.mx (C.M.-T.); carlos.pedrazardrg@uanl.edu.mx (C.P.-R.); lucio.veracb@uanl.edu.mx (L.V.-C.); jorge.ocampocnd@uanl.edu.mx (J.O.-C.); 2 Facultad de Ciencias Biológicas (FCB), Universidad Autónoma de Nuevo León (UANL), Av. Pedro de Alba s/n, San Nicolás de los Garza 66450, NL, Mexico; catalina.rivasmr@uanl.edu.mx

**Keywords:** natural products, *Hedeoma*, tuberculosis, non-tuberculous mycobacteria, antimycobacterial

## Abstract

Tuberculosis (TB) remains a major health problem worldwide, and the emergence of multi-resistant strains to first-line drugs has become the biggest obstacle to its treatment. On the other hand, the incidence of non-tuberculous mycobacteria (NTM) in humans has increased remarkably in recent years. The search for new and better treatments against mycobacterial infections is a constant at the global level. Hence, in this study, we propose to investigate the antimycobacterial effect of the extracts and major compounds of *Hedeoma drummondii* against clinical isolates of *Mycobacterium tuberculosis* and non-tuberculous mycobacteria: *M. abscessus*, *M. fortuitum*, *M. intracellulare*, and *M. gordonae*. To determine the antimycobacterial activity, a microdilution assay was used to establish the minimum inhibitory concentration (MIC) of the different strains of *Mycobacterium*. The methanolic extract presented the best activity against *M. tuberculosis*, inhibiting ten of the twelve strains analyzed at a concentration < 2500 µg/mL; meanwhile, the hexanic extract presented the best activity against non-tuberculous mycobacteria (NTM) by inhibiting eight of the ten strains studied at ≤625 µg/mL. Moreover, there is a strong positive correlation between the antimycobacterial activity of pulegone and the hexanic extract against non-tuberculous strains, so this compound could serve as a predictability marker against these types of microorganisms.

## 1. Introduction

Tuberculosis (TB) is a deadly and infectious lung disease caused by *Mycobacterium tuberculosis*, which has been threatening humanity for years and remains a major global health problem [1]. According to a 2018 global report by the World Health Organization (WHO), it was recorded that in 2017, there were an estimated 10 million new cases of TB. This is the equivalent to 133 cases per a 100,000 population [2]. The emergence of multidrug-resistant TB is mainly due to the inappropriate use of first-line anti-TB drugs, and the increased prevalence of such strains has become a major obstacle in TB treatment and a financial burden on the health sector [3]. As a result, there is an urgent need for new, cost-effective anti-TB drugs with different mechanisms of action and less opportunity to develop resistance [4].

On the other hand, non-tuberculous mycobacteria (NTM) are present on a wide diversity of surfaces, and their incidence in humans is increasing significantly worldwide [5]. It must be mentioned that progress in treating this type of mycobacteria has been slow due to the high number of NTM species and their clinical similarities, as well as low susceptibility to available antibiotics, which hinders correct diagnoses and the subsequent treatment [6]. One of the most important challenges of choosing an efficient treatment against NTMs has been the lack of correlation between in vitro susceptibility patterns and clinical responses [7]. In most non-tuberculous species, there are no evidence-based treatment recommendations, so physicians must make case-specific decisions on a case-by-case basis. Therefore, it is urgent to find a new strategy to combat mycobacterial infections [8].

**Figure 1:**
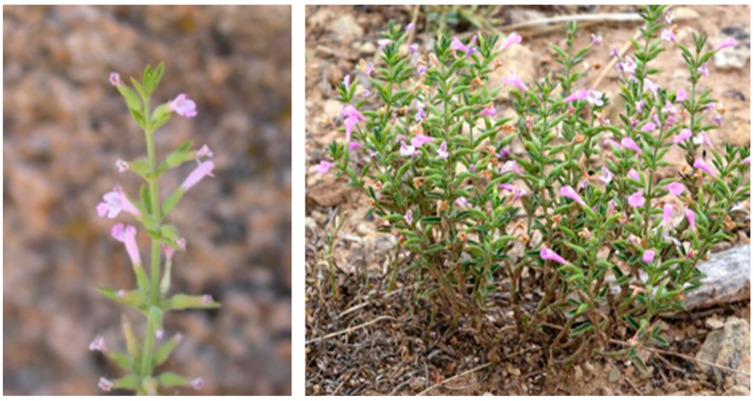
Inflorescences (**left**) and aerial parts (**right**) of *Hedeoma drummondii*.

Due to the resistance present in mycobacteria, a systematic search for compounds that are capable of dealing with this group of microorganisms has been carried out for years; natural products play an important role in this search, and many of these efforts are aimed at the study of medicinal plants, since they are used empirically for the treatment of various diseases, including respiratory diseases [9]. The research and standardization of medicinal plants has contributed to the development of phytopharmaceuticals, most of which have proven to be low cost and with low toxicity [10]. In relation to the statement above, in the northeastern region of Mexico, 234 species of medicinal plants used to cure respiratory diseases have been reported, highlighting the Lamiaceae family with 12 species [11]. It is within this aromatic family that *Hedeoma drummondii* is found. It can be described as a hairy perennial herb with an erect, mint-like shape up to 45 cm tall and the flowers are generally light to deep purple (Figure 1). The herb is used as a pleasant tea to relieve cold and cough in the north of Mexico and USA [12,13], and its extracts have already been reported to possess antibacterial activities, especially those of low polarity [14,15]. In these types of extracts and essential oils, the high content of the monoterpene pulegone stands out, while in the polar ones, rosmarinic and caffeic acids have been reported as the main bioactive compounds [15,16,17]. Therefore, the aim of this study was to assess the antimycobacterial activity from the crude extracts and major constituents from the Mexican aromatic plant *H. drummondii* against twelve strains of *Mycobacterium tuberculosis* and ten non-tuberculous strains.

## 2. Results

### 2.1. Determination of MIC in M. tuberculosis

**Figure 2:**
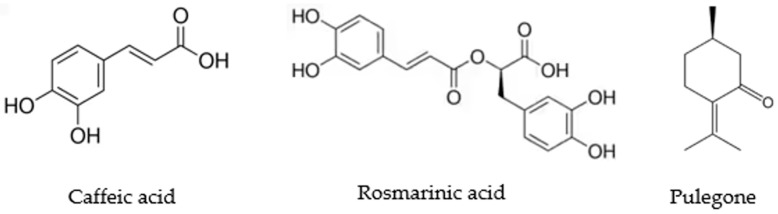
Structure of the main compounds from *H. drummondii* analyzed against *Mycobacterium* strains.

Table 1 shows the antimycobacterial effect of the extracts and major compounds (Figure 2) of *H. drummondii* against each of the twelve strains of *M. tuberculosis* in a microplate assay. Within the samples studied in the present work, the MIC of the hexanic extract against *M. tuberculosis* HU-LIID-D-159 was 312.5 µg/mL and that of the methanolic extract was 19.53 µg/mL for *M. tuberculosis* strains HU-LIID 168-99 and HU-LIID 142-99 and 39.06 µg/mL for strain HU-LIID 376-98. As for the major compounds, the MIC of pulegone stands out with an activity of 125 µg/mL in strains HU-LIID 90-99 and HU-LIID 428-98, while rosmarinic and caffeic acid presented activities of 125 µg/mL for strains HU-LIID 434-98, HU-LIID 168-99, HU-LIID 142-99, and HU-LIID 376-98.

### 2.2. Determination of MIC in NTM

Table 2 shows the antimycobacterial effect of the extracts and major compounds of *H. drummondii* against each of the ten NTM strains. When observing the inhibitory activity of the samples against NTMs, the hexanic extract obtained the best MIC concentrations, presenting values of 39.0625 µg/mL in *M. abscessus* LMMP and 78.125 µg/mL for *M. intracellulare* 989-3 and *M. intracellulare* 1105-1. On the other hand, the methanolic extract presented its best activity against *M. abscessus* 139-10 with an MIC of 78.125 µg/mL, followed by 312.5 µg/mL for *M. abscessus* LMMP, *M. fortuitum* 430R, *M. fortuitum* MLIID1, and *M. gordonae* A702 strains. While in the major compounds, pulegone was the one that obtained the best activity with MIC values of 15.625 µg/mL for *M. abscessus* 139-10 and *M. abscessus* LMMP as well as 31.25 µg/mL for *M. gordonae* A702, and in the case of rosmarinic acid, its activity was 250 µg/mL for most strains and caffeic acid stood out for its activity of 125 µg/mL against *M. abscessus* LMMP and *M. intracellulare* 142-09.

### 2.3. Pearson Correlation

The Pearson’s r correlation coefficient was used to measure the association of antimycobacterial activity between the extracts of *H. drummondii* and their respective compounds. The results indicated that against *M. tuberculosis* strains, the methanolic extract presented a weak correlation (r = 0.14) with caffeic acid and a moderate correlation (r = 0.23) with rosmarinic acid, while the hexanic extract presented a negative correlation with pulegone. However, when comparing the extracts and compounds of *H. drummondii* against the NTM strains, it was observed that the hexanic extract presented a strong correlation (r = 0.53) with pulegone, while the methanolic extract presented negative correlations with rosmarinic acid and caffeic acid.

## 3. Discussion

In the present work, the antimycobacterial activity of the extracts and major compounds of *H. drummondii* against clinical isolates of *M. tuberculosis* and non-tuberculous mycobacteria was demonstrated. The use of organic solvents of different polarities is common for the extraction of bioactive compounds, since those of low polarity (hexane, chloroform, petroleum ether, and so forth) are excellent for the extraction of compounds, such as terpenes, coumarins, quinones and related molecules. Meanwhile, the polar solvents (methanol, ethanol, acetone, and so on) are used for the extraction of flavonoids, phenolic acids, and saponins, among other molecules [18]. This could explain why in our results, the methanolic (polar) extract was better against *M. tuberculosis*, while the hexanic (non-polar) extract presented more favorable results against non-tuberculous mycobacteria since the compounds found in them are of different chemical nature. It is worth mentioning that, similar to our results against the *M. tuberculosis* H37Rv strain, the nonpolar (chloroform) extracts of *P. stellatum* and *O. integrifolia* were more active than the polar (methanolic) ones [19]; however, in our research, this effect was only demonstrated against that particular strain (Table 1). Analyzing the antimicrobial effect on as many strains as possible is important to find candidate treatments that can circumvent the mechanisms of drug resistance developed by them [20].

It was observed that the two extracts studied showed an effect against ten of the twelve strains of the *M. tuberculosis* tested. The best effect was shown by the methanolic extract with an MIC of 19.53 µg/mL against the strains 168-99 and 142-99. The analyzed *M. tuberculosis* strains had resistance to rifampicin, which is a first-line drug, by presenting an MIC greater than 1 μg/mL [21].

The efficacy of the methanolic extract of *H. drummondii* against various strains of *M. tuberculosis* may be partly due to the abundance of phenolic compounds present in it, as it has previously been suggested that these compounds, such as flavones (cycloartocarpin, artocarpin, chaplashin, morusin, cudraflavone B, and cudraflavone C), play a significant role in inhibiting the production of the mycolic acid that is involved in the formation of the mycobacterial cell wall in addition to nucleic acid and protease inhibition, which affects the growth and viability of *M. tuberculosis* [4,22].

In this research, it was found that rosmarinic and caffeic acid showed antituberculosis activity against 9 of the 12 strains tested (Table 1). In previous research, the antimycobacterial effect of caffeic acid has already been demonstrated against a diversity of clinical isolates of *M. tuberculosis*, showing an MIC in the range of 128 to 1024 µg/mL [23]. On the other hand, structural derivatives of rosmarinic acid amides have been studied for the inhibition of the enzyme UDP-galactopyranose mutase (UGM), which is essential for cell wall integrity and viability; its inhibition has been presented as a cost-effective strategy for the discovery of anti-tuberculosis compounds [24]. Likewise, it has been reported that the catechol group is found in both compounds (caffeic and rosmarinic acid) and is responsible for the antimicrobial activity shown by molecules possessing this functional group [25].

As for the effect against non-tuberculous mycobacteria (NTM), it was observed that the hexanic extract was the most active, inhibiting 8 of the 10 strains studied, with the most sensitive being *M. abscessus* LMMP, with an MIC of 39.06 µg/mL (Table 2). The effect of essential oils obtained from a diversity of Lamiaceae against non-tuberculous mycobacterial strains has been previously demonstrated, highlighting *Lavandula hybrida*, which showed an effect against *M. intracellulare* and *M. gordonae* with MICs of 3200 µg/mL and 6400 µg/mL, respectively, as well as *Salvia officinalis* with an MIC of 6400 µg/mL for *M. gordonae* [26]. In the same vein, antimicrobial activity against this group of mycobacteria has already been demonstrated by organic extracts obtained with low and medium polarity solvents: the chloroplast extract of the *Persea americana* seed showed MICs of 12.5, 25, 50, and 100 µg/mL for *M. smegmatis*, *M. abscessus*, *M. fortuitum*, and *M. chelonae*, respectively [27], while the hexanic extract obtained from *Juniperus communis* showed an MIC of 64 µg/mL against *M. aurum* [28].

The antimicrobial activity shown by the non-polar extract of *H. drummondii* (hexane) against non-tuberculous strains could be related to the fact that this group of mycobacteria contains glycopeptidolipids (GPLs) that are found in the cell envelope and contribute to biofilm formation, which is related to their spread, virulence, and resistance [29,30]. These GPLs can be inhibited by compounds of lipophilic terpene nature, such as monoterpenes [31].

In relation to the statement above, pulegone, one of the major monoterpenes of *H. drummondii*, showed antimycobacterial activity against 8 of the 10 NTM strains tested. The most sensitive strains were *M. abscessus* 139-10, LMMP, and *M. gordonae* A702 with MICs of 15.63 µg/mL and 31.25 µg/mL, respectively (Table 2). The effect of monoterpenes against non-tuberculous strains has been reported with the effect of carvacrol against *M. abscessus* and *M. fortuitum* with an MIC of 64 µg/mL [32], and eugenol against *M. kansasii, M. massilliense*, *M. chelonae*, *M. gordonae*, *M. smegmatis*, and *M. fortuitum* with MICs between 3.9 and 250 µg/mL [33], in addition to α-pinene, thymol, p-cymene, limonene, myrcene, and geraniol, with MICs between 250 and 500 µg/mL for *M. chelonae* [34].

In our research, it was found that the hexanic extract presented a strong correlation with pulegone (r = 0.53) with NTM strains [35], which means that pulegone could possibly serve as a predictability marker. Previous investigations have been conducted in the search for chemical markers of predictability, such as the correlation of the antioxidant activity of twelve species of the Zingiberaceae family with phenolic compounds [36] and the correlation of the α-glucosidase inhibitory activity of eight species of the Lamiaceae family with phenolic acids and flavonoids [37].

However, the presence of weak correlations between the bioactivity of major compounds and pure extracts does not necessarily mean a discouraging result for the formulation of phytopharmaceuticals. In previous research, it has been mentioned that the synergy between the minority compounds present in the extracts could bring benefits by decreasing the possibility of developing resistance against a particular compound [38]. This would be useful in the polar extract of *H. drummondii*, where there may be other compound(s) in addition to those analyzed with effects against the different mycobacteria analyzed.

## 4. Materials and Methods

### 4.1. Extraction and Isolation

*H. drummondii* (Lamiaceae) was collected in Allende, Nuevo León, México. The species was identified by Dr. Marcela Gonzalez Alvarez. A dried specimen was deposited in the ethno botanical collection of the Herbarium of Facultad de Ciencias Biológicas, Universidad Autónoma de Nuevo León, with the voucher number: 024244.

The aerial part of the plant was dried at room temperature, and 150 g of the dried plant was extracted by static maceration with hexane first and then with methanol at room temperature. The plant–solvent ratio was 1:10, changing the solvent every 24 h 3 consecutive times, and then the extract was filtered using Whatman No. 1 filter paper. The solvent was removed from the filtered product using a rotary evaporator with reduced temperature and pressure. The concentrated organic extracts were stored at 4 °C until use.

Methanolic extract of *H. drummondii* was partitioned with DCM:MeOH (1:1), and the polar partition was chromatographed in Sephadex LH-20 in order to obtain five fractions. The fraction 2 was rechromatographed by HPLC semi preparative to yield a mayor phenolic acid: caffeic acid (CafAc) and rosmarinic acid (RosAc), as previously described [19]. The monoterpene (*R*)-(+)-Pulegone was obtained commercially from Sigma-Aldrich (No. 376388).

### 4.2. Mycobacterial Strains and Culture Conditions

Ten clinical isolates of NTM and twelve strains of *M. tuberculosis* were provided by Laboratorio Interdisciplinario y de Investigación Dermatológica (LIID) belonging to the Hospital Universitario (HU) José E. González (Monterrey, Nuevo León, México). It should be mentioned that several of the *M. tuberculosis* strains used are resistant to one or more first-line treatment drugs. To differentiate the strains, the following key was used: HU-LIID, which alludes to the place where they were isolated and characterized.

All strains were identified to species level by biochemical tests and PCR restriction analysis of a 441-bp sequence of the hsp65 gene. The reference isolate *M. tuberculosis* H37Rv ATCC 27294 was obtained from the American Type Culture Collection (ATCC). The strains were activated from frozen stocks in Lowenstein–Jensen medium by incubation at 37 °C, CO_2_ 5% for 7 to 14 days.

### 4.3. Determination of the Minimum Inhibitory Concentration (MIC)

MIC of *M. tuberculosis* and NTM was evaluated using the 96-well plate microdilution technique according to CLSI [39]. For MIC evaluation in *M. tuberculosis*, solutions were prepared from the stocks of each sample with serial dilutions from 19.531 to 2500 µg/mL for the extracts and 3.906 to 500 µg/mL for the major compounds. Both cases used medium Middlebrook 7H9 supplemented with albumin–oleic acid–dextrose–catalase (OADC). While the antibiotic rifampicin was used as a control at concentrations from 0.25 to 32 µg/mL.

For each sample, 50 µL of each concentration was added horizontally in the microplate along with 50 µL of the strain to be evaluated, which had been previously adjusted to 1 on the McFarland scale, as well as a control well without treatment (*M. tuberculosis* strain in culture medium). This procedure was performed in duplicate and repeated for each strain in the study. The microplates were incubated at 37 °C for 5 d, and then Alamar Blue reagent was prepared in a 1:1 ratio with 10% Tween, adding 50 µL of the reagent to the wells, and the microplates were reincubated at 37 °C for 24 h [40]. MIC was defined as the lowest drug concentration that prevented a color change from blue to pink [41].

For MIC evaluation of NTM isolates, the same microdilution plate technique used for *M. tuberculosis* was followed; the media used are cation-adjusted Mueller–Hinton (CA-MHB) for *M. abscessus* and *M. fortuitum* and Middlebrook 7H9 medium supplemented with albumin- oleic acid- dextrose-catalase (OADC) for *M. intracellulare* and *M. gordonae*. While the control was performed with linezolid and imipenem antibiotics at serial dilutions from 0.25 to 32 µg/mL. Turbidity in the wells was measured after incubating the microplate at 37 °C for 72 h for *M. abscessus* and *M. fortuitum* strains and after 7 to 10 d for *M. intracellulare* and *M. gordonae* strains [42].

The MIC values obtained were analyzed by Pearson correlation to determine whether there was an association of antimycobacterial activity between the use of extracts and their respective compounds.

## 5. Conclusions

The organic extracts of *H. drummondii* possess antimycobacterial activities, with the hexanic one inhibiting 8 of the 10 NTM strains analyzed with an MIC between 39.0625 and 625 μg/mL, and the methanolic one inhibiting 10 of the 12 strains of *M. tuberculosis* with an MIC between 19.53 and 1250 μg/mL. The major compounds of *H. drummondii* possess antimycobacterial effects, with pulegone standing out by inhibiting 11 of the 12 *M. tuberculosis* strains with MICs between 125 and 500 μg/mL, as well as 8 of the 10 NTM strains tested with MICs between 15.625 and 500 μg/mL. A strong correlation was observed between the hexanic extract and the pulegone compound with its effect against non-tuberculous mycobacteria (r = 0.53), which opens the possibility of it being used as a marker of predictability for the antimycobacterial activity of the non-polar extract.

The current study indicates that crude extracts of *H. drummondii* can be explored for potential leads in antimycobacterial therapy. The pharmacokinetics and pharmacodynamics of these extracts need to be studied in physiologically more complex models to demonstrate their intracellular effect before they could be actually taken up further as complementary and/or alternative therapy.

## Figures and Tables

**Table 1:** Antimycobacterial activity in the extracts and major compounds of *H. drummondii* against *M. tuberculosis*. In bold, we show those extracts with activity at <500 µg/mL.

*M. tuberculosis* Strains	HexEx	MetEx	Pulegone	RosAc	CafAc	* Rifampicin
HU-LIID 159	312.5	625	500	500	500	16
HU-LIID 386-98	625	1250	>500	>500	>500	32
HU-LIID 41-99	>2500	>2500	500	>500	>500	16
HU-LIID 90-99	2500	**156.25**	125	250	125	8
HU-LIID C-131	>2500	625	500	500	500	16
HU-LIID 428-98	1250	>2500	125	>500	>500	32
HU-LIID F-353	1250	**312.5**	250	250	250	16
HU-LIID 434-98	2500	1250	500	125	125	8
HU-LIID 168-99	1250	**19.53**	250	125	125	8
HU-LIID 142-99	1250	**19.53**	500	125	125	8
HU-LIID 376-98	1250	**39.06**	500	125	250	4
ATCC H37Rv	**156.25**	625	500	500	250	2

Data in µg/mL. HexEx: hexanic extract; MetEx: methanolic extract; RosAc: rosmarinic acid; CafAc: caffeic acid. * Reference drug.

**Table 2:** Antimycobacterial activity in the extracts and major compounds of *H. drummondii* against non-tuberculous mycobacteria. In bold, we show those extracts with activity at < 500 µg/mL.

*Mycobacterium* Strains	HexEx	MetEx	Pulegone	RosAc	CafAc	* Linezolid	* Imipenem
*M. abscessus* 139-10	**312.5**	**78.125**	15.625	250	250	4	4
*M. abscessus* LMMP	**39.06**	**312.5**	15.625	250	125	4	4
*M. abscessus* MNT2	625	>2500	62.5	250	> 500	2	16
*M. fortuitum* 430R	>2500	**312.5**	500	>500	>500	32	4
*M. fortuitum* MLIID1	>2500	**312.5**	>500	>500	>500	32	8
*M. intracellulare* 989-3	**78.125**	625	250	250	250	8	8
*M. intracellulare* 142-09	**156.25**	>2500	250	250	125	16	32
*M. intracellulare* 1105-1	**78.125**	>2500	>500	> 500	500	32	16
*M. gordonae* 621R	**312.5**	>2500	250	> 500	250	8	16
*M. gordonae* A702	**156.25**	**312.5**	31.25	250	250	8	32

Data in µg/mL. HexEx: hexanic extract; MetEx: methanolic extract; RosAc: rosmarinic acid; CafAc: caffeic acid. * Reference drug.

## Data Availability

Not Applicable.
